# Supplementary material for: Evolution of the Tumor Microenvironment toward Immune-Suppressive Seclusion during Brain Metastasis of Breast Cancer: Implications for Targeted Therapy
Source: Cancers (Basel). 2021 Sep 29;13(19):4895. doi: 10.3390/cancers13194895 (PMC8507988; doi:10.3390/cancers13194895)
Supplement: Supplementary file 1 [file cancers-13-04895-s001.zip › cancers-1387773-supplementary.pdf]

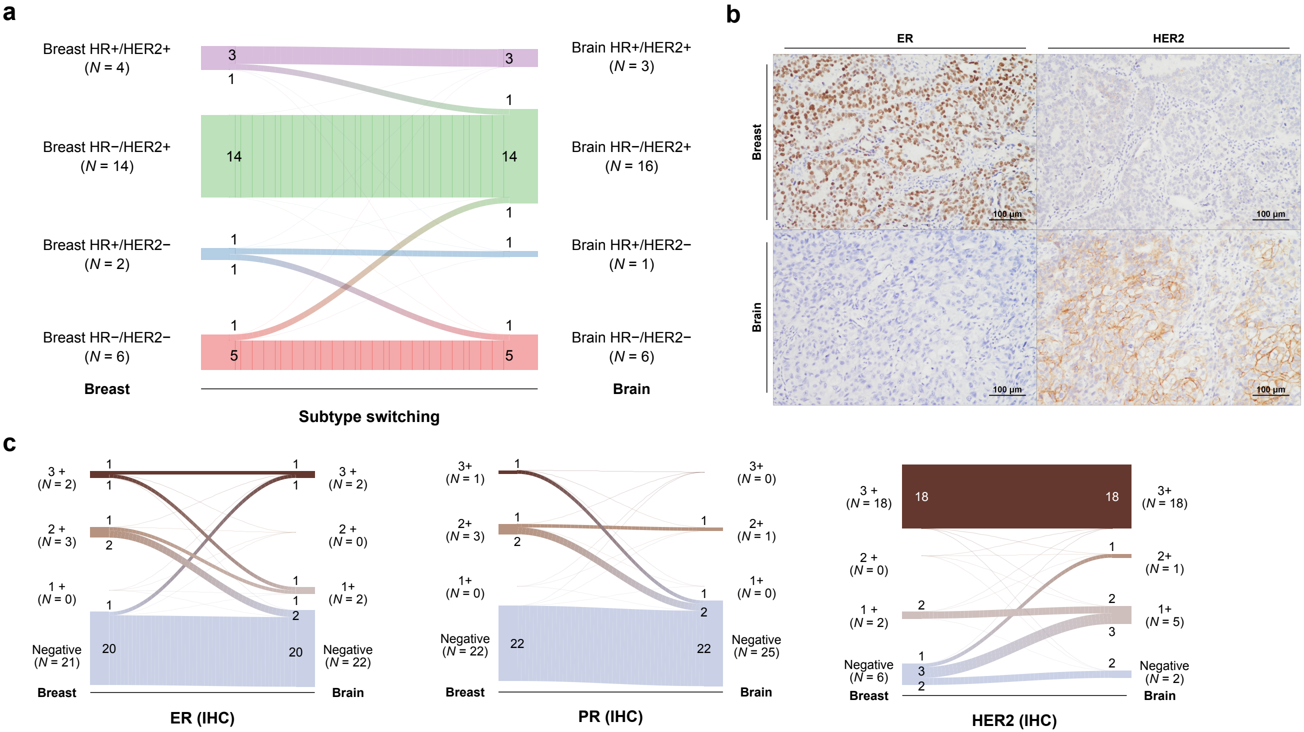

Supplementary Figure S1. (a) River plot showing molecular subtype switching in patient-matched cases during brain metastasis (BM). Width of the bands indicates the number of patients crossing over. (b) One sample case showing decreased estrogen receptor (ER) expression and enhanced human epidermal growth factor receptor 2 (HER2) expression during BM with representative immunohistochemistry (IHC) histograms from primary breast cancer (top) and BM (bottom). From left to right, IHC staining for ER and HER2 (original magnification, ×200). (c) River plot showing the IHC expression changes of ER (left), progesterone receptor (PR) (middle), and HER2 (right) in patient-matched cases during BM. Width of the bands indicates the number of patients crossing over.

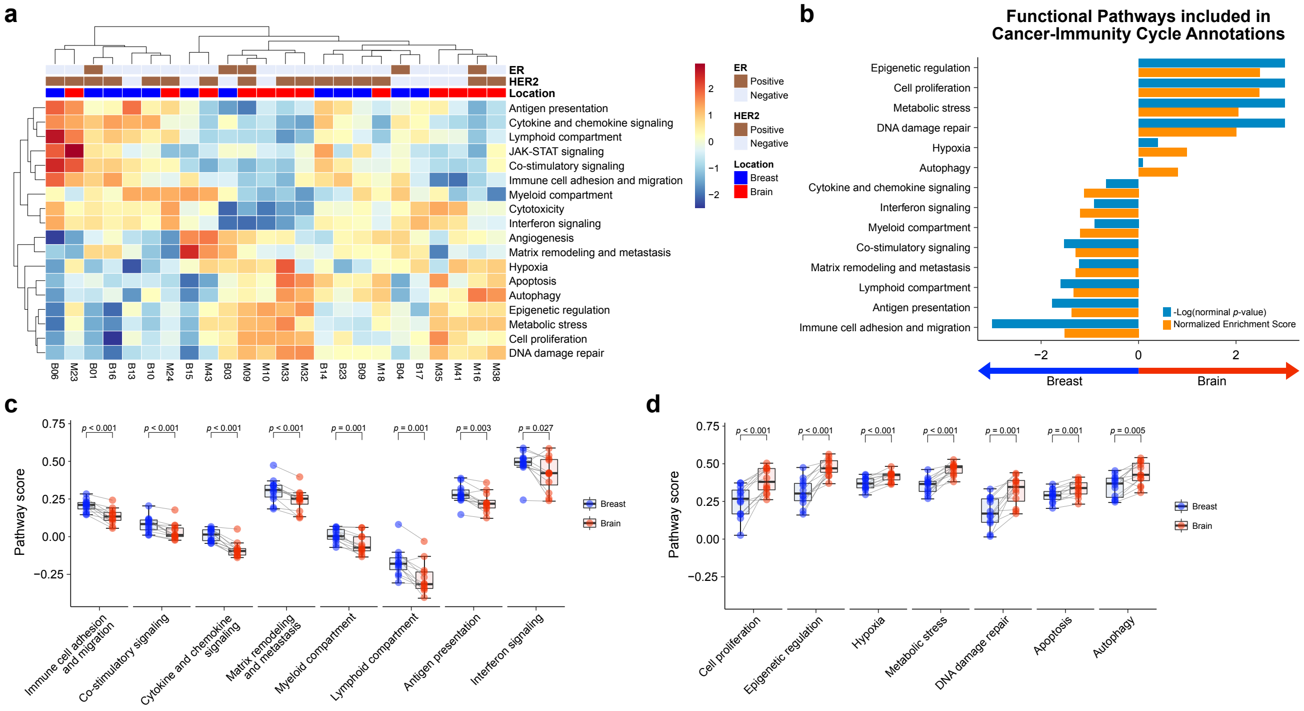

Supplementary Figure S2. (a) Pathway scores of functional pathways included in the cancer-immunity cycle annotation gene set of the nCounter® PanCancer IO 360™ Panel, calculated with single-sample GSEA (ssGSEA) and visualized as a heatmap. Scores were Z-transformed and displayed on the same scale. (b) Gene set enrichment analysis of primary breast cancer *versus* brain metastasis (BM) plotted using the gene sets for the functional pathways included in cancer-immunity cycle annotation. (c–d) Pairwise box plots of functional pathways included in cancer-immunity cycle annotation according to location, showing the sets of pathway scores downregulated in BM (c) and those upregulated in BM (d).

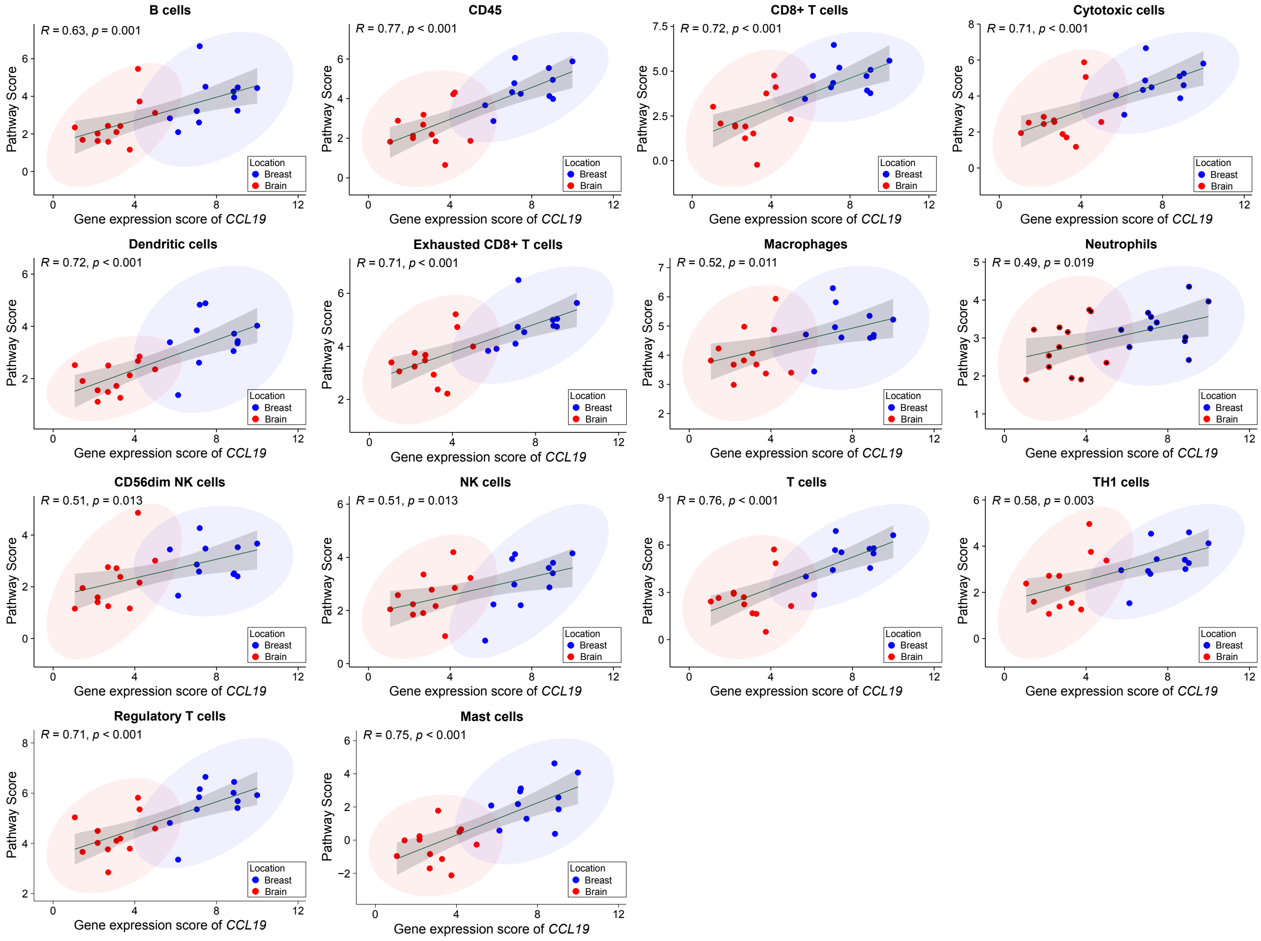

Supplementary Figure S3. Linear correlation analysis of *CCL19* expression and 14 immune-profile signatures of PanCancer IO 360. The 14 immune-profile signatures were positively correlated with *CCL19* expression.

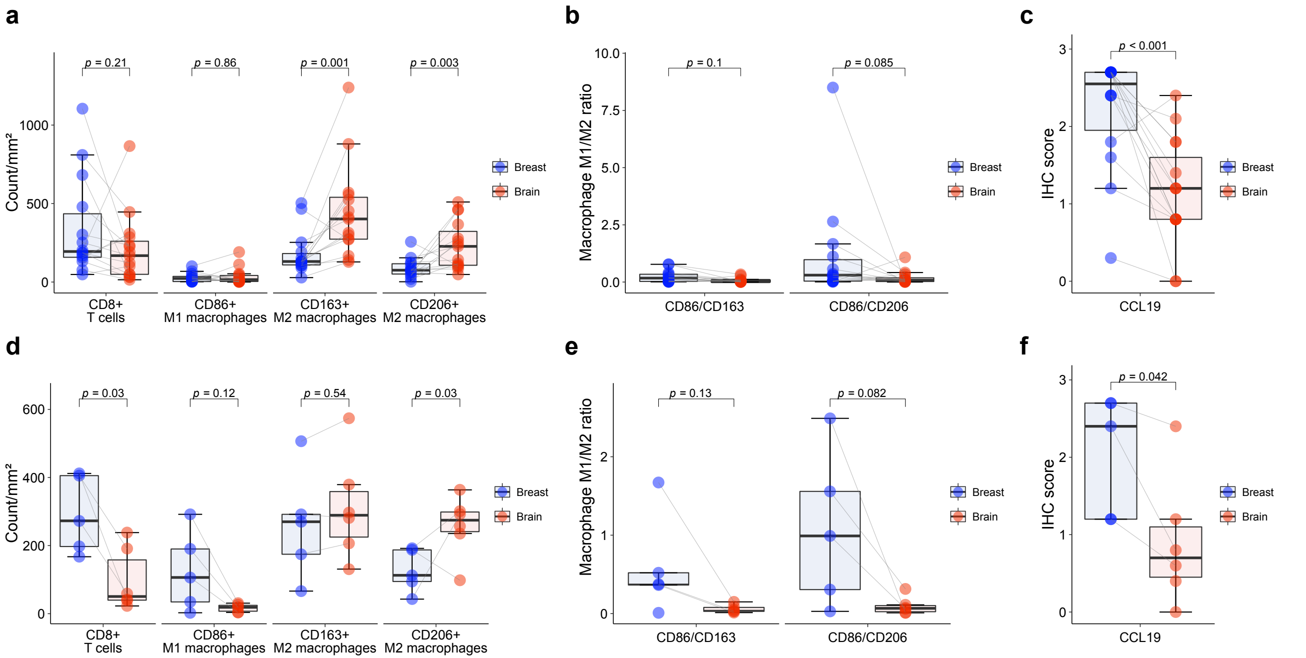

Supplementary Figure S4. Immunohistochemical (IHC) analysis of immune cell profiles in primary breast cancer and brain metastasis (BCBM) by subtype of breast cancer. (a–c) IHC analysis in human epidermal growth factor receptor 2 (HER2) positive type. Pairwise box plots of counts of CD8+ cells, CD86+ cells, CD163+ cells, and CD206+ cells according to tumor location (a), pairwise box plots showing the relative proportions of CD86+ cells per CD163+ cells or CD206+ cells according to tumor location (b), pairwise box plot of CCL19 expression in HER2 positive type of primary breast cancer and BM samples according to tumor location (c). (d–f) IHC analysis in triple negative type. Pairwise box plots of counts of CD8+ cells, CD86+ cells, CD163+ cells, and CD206+ cells according to tumor location (d), pairwise box plots showing the relative proportions of CD86+ cells per CD163+ cells or CD206+ cells according to tumor location (e), pairwise box plot of CCL19 expression in triple negative type of primary breast cancer and BM samples according to tumor location (f).

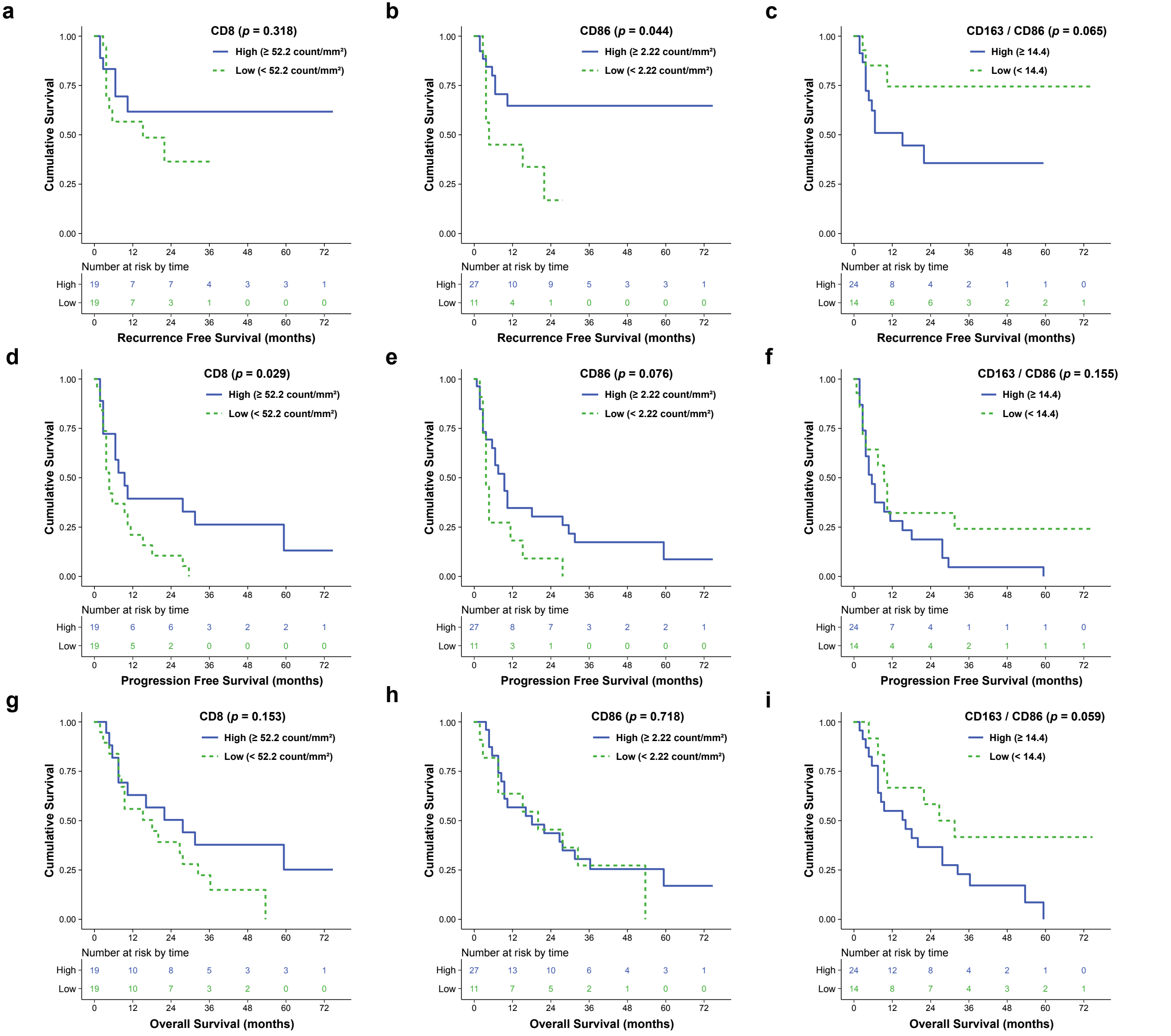

Supplementary Figure S5. Kaplan-Meier survival analyses according to immune cell density. (a–c) Recurrence-free survival analysis according to CD8+ cell count (a), the ratio of CD86+ cells to CD163+ cells (b), and the ratio of CD86+ cells to CD206 cells (c). (d–f) Progression-free survival analysis according to CD8+ cell count (d), the ratio of CD86+ cells to CD163+ cells (e), and the ratio of CD86+ cells to CD206 cells (f). (g–i) Overall survival analysis according to CD8+ cell count (g), the ratio of CD86+ cells to CD163+ cells (h), and the ratio of CD86+ cells to CD206 cells (i). P-values by log-rank test.
